# Role of gastric bypass surgery in control of blood sugar in obese uncontrolled type 2 diabetic patients

**DOI:** 10.1186/s13098-024-01281-4

**Published:** 2024-02-19

**Authors:** Hala Khalf Allah El-Shreef, Osama Yaseen Mostafa Taha, Heba Ahmed abd El Hafeez, Amal Ibrahim Abd El-Rheem Abo Shoka

**Affiliations:** 1 Department of Internal Medicine, Assiut University Hospital, Faculty of Medicine, Assiut University, Al-Azhar Street, Taksim Soliman El-Hakim, Assiut, Egypt; 2 Department of Plastic Surgery, Assiut University Hospital, Faculty of Medicine, Assiut University, Assiut, Egypt; 3 Department of Clinical Pathology, Assiut University Hospital, Faculty of Medicine, Assiut University, Assiut, Egypt; 4 Department of Internal Medicine, Assiut University Hospital, Faculty of Medicine, Assiut University, Assiut, Egypt

**Keywords:** Diabetes mellitus, Obesity, Endocrinology, Gastric bypass surgery

## Abstract

**Background and goal:**

The pandemic of the twenty-first century is diabetes. Both type 2 diabetes mellitus and obesity pose severe problems for public health. Despite significant improvements in diagnosing and managing both conditions, diabetes mellitus remains poorly controlled, and diabetic complications are more common than ever. Internists have discovered over the past 20 years that obese people with type 2 diabetes who have gastric bypass surgery to shed weight have improved glycemic control. Thus, interventional diabetology has a growing significance in patients’ ability to reverse type 2 diabetes mellitus. We want to evaluate the impact of gastric bypass on blood sugar regulation and look for potential causes.

**Patients and methods:**

Between 2018 and 2020, a prospective interventional study was carried out. Ninety patients in total were enrolled in the trial. The two patient groups (A and B) contained 45 obese T2DM patients with a body mass index (BMI) of over 35 kg/m^2^. Group B received its antidiabetic medications, either oral hypoglycemic or insulin, while Group A underwent gastric bypass surgery. Each patient underwent a comprehensive history review and clinical assessment. Both groups had their HA1c and blood sugar levels measured; group A had their insulin, GLP-1, and HOMA-IR (Homeostasis Model Assessment for Insulin Resistance) levels calculated at time O and one year later.

**Results:**

The demographic differences between the two study groups were negligible. After a one-year follow-up, group A had significantly lower anthropometric measurement data for BMI and waist circumference (cm), lipid profile data for triglycerides, total cholesterol, and LDL levels, and systolic and diastolic blood pressure. Fasting plasma glucose and HbA1c, two metrics of glucose metabolism, significantly decreased in group A. Regarding indicators of glucose metabolism, there was a drop in fasting insulin level and HOMA-IR and an increase in GLP1 level in the gastric bypass group.

**Conclusion:**

As a result of improving all indicators, gastric bypass is an effective treatment for patients with uncontrolled T2DM. Future research that is confirmed is needed.

## Introduction

The pandemic of the twenty-first century is diabetes. Both type 2 diabetes and obesity pose severe problems for public health. The connection between the two diseases is crucial since type 2 diabetes risk is significantly increased by fat. About 95% of cases of diabetes in the US in 2013 were type 2. Over 90% of people with type 2 diabetes are obese or overweight. Though type 2 diabetes is increasingly prevalent in young individuals, it often manifests in middle or older age. As type 2 diabetes and obesity rates rise, so do societal and direct patient care expenses [1]. The life expectancy of people with type 2 diabetes is ten years lower than those without the disease. Premature death is commonly caused by type 2 diabetes. It causes numerous chronic health issues, such as eye, foot, and chronic renal illnesses, as well as macrovascular and microvascular consequences [2].

Patients with type 2 diabetes who are obese can benefit from modest weight loss, even 5% of total body weight. Internists have discovered over the past 20 years that obese T2DM patients with gastric bypass surgery for weight loss exhibit improved glycemic control. Alterations to the gastrointestinal tract architecture alter the intrinsic regulating mechanism of glucose homeostasis. Some bariatric procedures are available for Roux-en-Y gastric bypass, sleeve gastrectomy, gastric banding, and biliopancreatic diversion [3]. Anatomic changes following laparoscopic and endoscopic interventions lead to changes in the neuroendocrine hormone profiles of the GI tract, which mediate additional metabolic effects. For this reason, interventional diabetology is becoming increasingly crucial in T2DM patients experiencing remission [4].

## Patients and methods

### Study design and setting

A prospective interventional study was conducted at Assuit University Hospital and private clinics between 2018 and 2020.

### Selection criteria

The enrolled patients with uncontrolled diabetes type 2 based on laboratory data. Patients with one or more of the following criteria were excluded: Patients with type I diabetes mellitus, Patients with BMI < 30 kg/m^2^, and Patients who refused to participate in the study.

### Participants

Ninety patients in total were enrolled in the trial. Two groups of patients, A and B, contained 45 obese T2DM patients with a body mass index (BMI) of over 35 kg/m^2^. Group B received their antidiabetic medications, either oral hypoglycemic or insulin, while Group A underwent gastric bypass surgery. The patients included in the study should have the following criteria: (1) Biochemical proof of the type should be present in the study’s participants. (2) The following diagnostic criteria confirm diabetes: 126mg/dl or higher in fasting plasma glucose. b) A plasma glucose level of 200 mg/dl or higher two hours after a meal. (1) HBAIC greater than 7.1%. (2) Diabetes has been present for 5 years. (3) Despite receiving effective clinical care and antidiabetic therapy, poor glycemic control (HBAIC 7%). (4) BMI > 35 kg/m_2_.

Both groups were subjected to the following laboratory tests at both time points (0 and 1 year): (1) A thorough blood count. (2) Blood sugar levels following a meal. (3) Serum creatinine and blood urea. (4) The INR, prothrombin time, and concentration. (HBAIC) Glycated hemoglobin. (6) Lipid composition. (7) Microalbuminuria. For group A, estimates of the levels of insulin and GLP-1 were made at time O and one year later. (8) For group A, HOMA-IR, or homeostasis model assessment for insulin resistance, was determined using the following formulas:

$$ {\text{HOMA}} - {\text{IR}} = \frac{{{\text{Glucose}} \times {\text{Insuling}}}}{22.5} $$

Glucose by mmol and insulin by mu/l. Patients in group B were instructed to continue their medical therapy and try diet control measures and lifestyle modification to reach their glycemic target.

### Ethics approval and consent to participate

The Ethics Review Board of the Faculty of Medicine at Assiut University approved the study methodology, and all subjects provided written informed permission per the Helsinki Declaration.

### Statistical analysis

SPSS software, version 25, from Chicago, IL, USA, was used to analyze all data. The Student T-test was used to compare continuous variables, and the Chi-square test was used to compare categorical variables in both groups. We used the Shapiro-Wilkes test to determine whether the data were normal before statistical analysis. A p-value of 0.05 was considered significant.

## Results

### Demographic data in the two studied groups

Demographic data in study groups showed a non-significant difference regarding age, gender, residence, work, and smoking (P > 0.05) (Table 1).

**Table 1:** Demographic data in the study groups

Items	Group		P-value
	Gastric bypass (n = 45)	Antidiabetic treatments (n = 45)	
Age Mean ± SD	49.22 ± 5.63	47.82 ± 5.40	0.232

		n	%	n	%	
Gender	Male	14	31.1	15	33.3	0.822
	Female	31	68.9	30	66.7	
Residence	Urban	30	66.7	27	60.0	0.512
	Rural	15	33.3	18	40.0	
Work	Work	26	57.8	19	42.2	0.140
	Not working	19	42.2	26	57.8	
Smoking		16	35.6	23	51.1	0.136

Values are presented as mean ± SD or number (%), *P value is significant if < 0.05

### History data in study groups

History data in study groups showed that there was a non-significance difference between gastric bypass and antidiabetic treatment groups regarding the duration of DM (years), type of treatment, antihypertensive drug, and hypolipidemic drug (P > 0.05) (Table 2).

**Table 2:** History data in the study groups

Items	Group				P value
	Gastric bypass (n = 45)		Antidiabetic treatments (n = 45)		
	Mean	SD	Mean	SD	
Duration of DM (years)	9.27	1.43	12.37	1.06	0.545

		n	%	n	%	
Type of treatment	Oral antidiabetic drugs	30	66.7%	27	60.0%	0.508
	Insulin	10	22.2%	9	20.0%	
	Oral antidiabetic drugs + insulin	5	11.1%	9	20.0%	
Antihypertensive drug		34	75.6%	36	80.0%	0.612
Hypolipidemic drug		34	75.6%	30	66.7%	0.352

Values are presented as mean ± SD or number (%)

*P value is significant if < 0.05

### Anthropometric measurements in study groups

Anthropometric measurement data were measured in study groups before treatments and after 1 year, showing that there was a significant difference (P < 0.05) between both groups after 1 year regarding BMI and waist circumference (cm). In the surgery group, BMI levels decreased after 1 year (P < 0.01) compared with baseline. Waist circumference (cm) was decreased after 1 year post-surgery compared with baseline. In the control group, BMI and waist circumference (cm) before treatments and after 1 year did not change between visits (Table 3).

**Table 3:** Anthropometric measurement data in the study groups

Variables		Group				P-value between group
		Gastric bypass (n = 45)		Antidiabetic treatments (n = 45)		
		Mean	SD	Mean	SD	
Height		159.27	6.19	157.93	5.41	0.318
Weight		112.67	10.57	108.87	11.22	0.051
BMI	Before treatment	44.57	5.15	43.85	5.87	0.490
	After 1 year	29.98	4.40	42.86	4.93	< 0.001*
	Change (Δ)	− 14.60	1.60	− 0.99	6.10	
P value within a group		< 0.001*		0.094		
Waist circumference (cm)	Before treatment	110.68	6.10	108.81	4.86	0.112
	After 1 year	89.08	6.63	108.91	5.03	< 0.001*
	Change (Δ)	− 21.60	1.83	0.10	1.70	
P value within a group		< 0.001*		0.778		

Values are presented as mean ± SD or number (%), *P value is significant if < 0.05

### Lipid profile data in the study groups

Before and after treatments, study groups’ lipid profiles were evaluated, and the results revealed a significant difference (P 0.05) between the two groups in terms of triglycerides (mg/dl), cholesterol (mg/dl), LDL (mg/dl), and HDL (mg/dl) levels. Triglycerides (mg/dl), cholesterol (mg/dl), and LDL (mg/dl) levels in the surgical group dropped after a year (P 0.01) in comparison to the starting point. After a year, HDL (mg/dl) increased (P 0.01) compared to the starting point. The lipid profile in the control group remained unchanged between visits before and after the first year of therapy (Table 4).

**Table 4:** Lipid profile data in the study groups

Variables		Group				P-value between group
		Gastric bypass (n = 45)		Antidiabetic treatments (n = 45)		
		Mean	SD	Mean	SD	
Triglycerides (mg/dl)	Before treatment	225.22	16.94	216.47	20.91	0.654
	After 1 year	167.42	23.89	187.51	25.49	< 0.001*
	Change (Δ)	− 57.80	14.14	− 18.96	10.23	
P value within a group		− < 0.001*		< 0.001*		
Cholesterol (mg/dl)	Before treatment	197.53	16.75	191.82	14.20	0.085
	After 1 year	167.60	21.07	187.02	14.96	< 0.001*
	Change (Δ)	− 29.93	11.02	− 4.80	9.62	
P value within a group		< 0.001*		< 0.001*		
LDL (mg/dl)	Before treatment	102.40	14.61	97.24	14.06	0.092
	After 1 year	92.22	12.17	95.18	14.41	0.296
	Change (Δ)	− 10.18	9.65	− 2.07	3.21	
P value within a group		< 0.001*		< 0.001*		
HDL (mg/dl)	Before treatment	42.22	5.68	42.78	5.26	0.632
	After 1 year	52.80	7.49	46.67	5.13	< 0.001*
	Change (Δ)	10.58	5.17	3.89	5.04	
P value within a group		< 0.001*		< 0.001*		

Values are presented as mean ± SD or number (%)

*P value is significant if < 0.05

### Glucose metabolism parameters in study groups

Glucose metabolism parameters were measured in study groups before treatments and after 1 year, showing that there was a significant difference (P < 0.05) between both groups after 1 year regarding Fasting plasma glucose (mg/dl) and HbA1c %**.** In the surgery group, Fasting plasma glucose (mg/dl) and HbA1c % decreased after 1 year (P < 0.01) compared with baseline (Table 5).

**Table 5:** Glucose metabolism parameters in the study groups

Variables		Group				P-value between group
		Gastric bypass (n = 45)		Antidiabetic treatments (n = 45)		
		Mean	SD	Mean	SD	
Fasting plasma glucose (mg/dl)	Before treatment	142.78	16.29	146.04	14.54	0.318
	After 1 year	91.29	15.63	133.27	15.22	< 0.001*
	Change (Δ)	− 51.49	14.41	− 12.78	2.13	
P value within a group		< 0.001*		< 0.001*		
HbA1c %	Before treatment	10.21	1.90	9.97	1.77	0.537
	After 1 year	6.93	1.25	8.73	1.68	< 0.001*
	Change (Δ)	− 2.53	1.65	− 1.24	0.32	
P value within a group		< 0.001*		− < 0.001*		

Values are presented as mean ± SD or number (%)

*P value is significant if < 0.05

### Other glucose metabolism biomarkers in the gastric bypass group

Fasting insulin (mIU/L) and HOMA-IR were measured in group A only before treatments. After 1 year, it was shown that there was a significant difference (P < 0.05) between the two measurements after 1 year. Both of them decreased after one year of the surgery (Table 6).

**Table 6:** Other glucose metabolism biomarkers in the gastric bypass group

Variables		Gastric bypass (n = 45)	
		Mean	SD
Fasting insulin (mIU/L)	Before treatment	23.29	10.08
	After 1 year	11.38	4.84
	Change (Δ)	− 11.91	8.29
P value within a group		< 0.001*	
HOMA-IR	Before treatment	8.29	3.69
	After 1 year	3.12	1.49
	Change (Δ)	− 5.17	3.04
P value within a group		− < 0.001*	

Values are presented as mean ± SD or number (%)

*P value is significant if < 0.05

### GLP-1 30-min peak (pmol/l) level in the gastric bypass group

GLP-1 30-min peak (pmol/l) level was measured in group A only before treatments and after 1 year showed that there was a significant difference (P < 0.05) between the two measurements after 1 year. GLP-1’s 30-min peak (pmol/l) level increased one year after the surgery (Table 7).

**Table 7:** GLP-1 30-min peak (pmol/l) level in the gastric bypass group

Variables		Gastric bypass (n = 45)	
		Mean	SD
GLP-1 30-min peak (pmol/l)	Before treatment	8.89	2.64
	After 1 year	41.79	6.89
	Change (Δ)	32.90	6.10
P value within a group		< 0.001*	

Values are presented as mean ± SD or number (%)

*P value is significant if < 0.05

## Discussion

With 90 participants, this prospective interventional study got underway. Two groups of patients, A and B, contained 45 obese T2DM patients with a body mass index (BMI) of over 35 kg/m^2^. We then assessed their patient characteristics, clinical data, comorbidities, and laboratory results. Group A got gastric bypass, while group B received antidiabetic therapies, either oral hypoglycemic medicines or insulin.

In keeping with Elder and Wolfe (2007) [5], the participants in the present study were T2DM patients with a body mass index (BMI) > 35 kg/m^2^. Our results were consistent with those of Davies et al. (2004) in that patients who underwent bypass surgery had lower BMIs (− 14.60 vs. − 0.99) and smaller waist circumferences (− 21.60 vs. 0.10) than patients who underwent medical treatment [6].

Systolic blood pressure (SBP) was decreased by (− 20.98) in the current study’s bypass surgery patients, compared to (− 2.69) in the medical treatment patients, and diastolic blood pressure (DBP) was decreased by (− 15.67) in the bypass patients, compared to (− 0.44) in the medical treatment patients. These outcomes are consistent with a study on 120 patients by Ikramuddin et al. (2013) [7]. 60 of the 120 patients got bypass surgery. One year after the experiment’s start, LDL decreased in patients who had bypass surgery by (− 10.18) compared with baseline, while it decreased by (− 2.07) in patients receiving medicinal treatment. Compared to baseline after 1 year, HDL levels increased in patients who underwent bypass surgery by 10.58 and in patients who received medicinal treatment by 3.89, respectively.

These findings were in line with those of Nosso et al. (2016) [8], who conducted a prospective study on 19 obese T2DM patients, of whom nine underwent bypass surgery and ten received medical treatment for two years. They estimated the lipid profile before and after the two years and found that triglycerides decreased the P-value (0.05) while HDL cholesterol increased. However, LDL fasting cholesterol decreased in patients who underwent bypass surgery instead of those who received medical treatment.

In the current study, patients who underwent bypass surgery experienced a significant decrease in fasting plasma insulin of (− 11.91), a significant decrease in HbA1c of (− 2.53), and a significant decrease in HbA1c of (− 1.24) when compared to baseline levels in patients who received medical treatment.

Our findings differed from those of Nosso et al. (2016) [8], who found that the bypass group had higher fasting plasma glucose and insulin levels than the medical therapy group (Nosso et al., 2016). In the current study, the HOMA -IR level significantly decreased; it started at 8.29 and, after one year, had dropped to 3.12 with a reduction rate of 5.17 (P-value 0.001). These findings were supported by Zhu et al. (2017) [9], who investigated eight very obese patients who underwent LGB and found that HOMA-IR decreased to a stable level in the third month following surgery and was strongly associated with complete remission.

According to the current study, bypass surgery patients had a substantial rise in GLP-1 levels (32.90) compared to baseline. This result was consistent with Shah and Laferrère’s (2016) findings [10], according to which bariatric surgery can boost post-prandial GLP-1 levels in both T2D-obesity and T2D-free obese patients by up to threefold before surgery and by approximately eightfold following gastric bypass.

## Conclusion

Due to improvements in all parameters, a significant decrease in BMI and increased weight loss, a significant decrease in blood pressure (systolic and diastolic), and a significant decrease in lipid profile (triglycerides, LDL, and HDL), bariatric surgery is an effective treatment for T2DM patients. Future research is necessary to verify these results.

## Data Availability

The data that support the findings of this study are available on request from the corresponding author

## References

[1] Černý V (2016). Guidelines for the management of severe traumatic brain injury, fourth edition. Anesteziol a Intenziv Med.

[2] Kuipers EJ, Grady WM, Lieberman D, Seufferlein T, Sung JJ, Boelens PG, et al. Colorectal cancer. Nat Rev Dis Prim. 2015;1. 10.1038/nrdp.2015.65 PMC4874655 27189416

[3] Sung KC, Wild SH, Byrne CD (2013). Resolution of fatty liver and risk of incident diabetes. J Clin Endocrinol Metab.

[4] Sims EK, Carr ALJ, Oram RA, DiMeglio LA, Evans-Molina C (2021). 100 years of insulin: celebrating the past, present and future of diabetes therapy. Nat Med.

[5] Elder KA, Wolfe BM (2007). Bariatric surgery: a review of procedures and outcomes. Gastroenterology.

[6] Ballantyne GH, Svahn J, Capella RF, Capella JF, Schmidt HJ, Wasielewski A (2004). Predictors of prolonged hospital stay following open and laparoscopic gastric bypass for morbid obesity: body mass index, length of surgery, sleep apnea, asthma and the metabolic syndrome. Obes Surg.

[7] Ikramuddin S, Korner J, Lee WJ, Connett JE, Inabnet WB, Billington CJ (2013). Roux-en-Y gastric bypass vs intensive medical management for the control of type 2 diabetes, hypertension, and hyperlipidemia: the diabetes surgery study randomized clinical trial. JAMA.

[8] Nosso G, Griffo E, Cotugno M, Saldalamacchia G, Lupoli R, Pacini G (2016). Comparative effects of roux-en-y gastric bypass and sleeve gastrectomy on glucose homeostasis and incretin hormones in obese type 2 diabetic patients: a one-year prospective study. Horm Metab Res.

[9] Zhou M, Wang H, Zeng X, Yin P, Zhu J, Chen W (2019). Mortality, morbidity, and risk factors in China and its provinces, 1990–2017: a systematic analysis for the Global Burden of Disease Study 2017. Lancet Elsevier.

[10] Shah A, Laferrère B (2017). Diabetes after bariatric surgery. Can J Diabetes.

